# Incidental intraprostatic paraganglioma: Case report and review of the literature

**DOI:** 10.1016/j.eucr.2026.103444

**Published:** 2026-04-20

**Authors:** Diego Gonzalez, Francis Ryan, Kris Kokoneshi, Sam Kwon, Liza Khutsishvili, Fiona Wardrop, Jacob Weiss, Shawn Reginauld, Maher Ali, Abiye Kassa, Michael Whalen

**Affiliations:** a George Washington University School of Medicine and Health Sciences, Washington, DC, USA; b Department of Pathology, George Washington University School of Medicine and Health Sciences, Washington, DC, USA; c Department of Urology, George Washington University School of Medicine and Health Sciences, Washington, DC, USA

## Abstract

Intraprostatic paraganglioma (PGL) is a rare neuroendocrine tumor arising from chromaffin cells and poses diagnostic and management challenges. We report a 63-year-old man with Grade Group 3 prostate adenocarcinoma who underwent robotic radical prostatectomy and was incidentally found to have a 1.5 mm intraprostatic PGL on final pathology. The patient had no catecholamine-related symptoms, and postoperative plasma metanephrines were normal, consistent with a nonfunctional tumor. Immunohistochemical staining supported the diagnosis. The patient elected surveillance and remains asymptomatic. This case highlights the variable presentation of prostatic PGL and emphasizes the role of pathologic evaluation, biochemical testing, and individualized management strategies.

## 1 Introduction

Neuroendocrine tumors originate from chromaffin cells of the autonomic nervous system and can be categorized based on their origin and location. An ectopic pheochromocytoma, or paraganglioma (PGL), is a catecholamine-secreting tumor located outside of the adrenal medulla. Head and neck PGLs are non-catecholamine-secreting tumors of parasympathetic origin and account for approximately 70% of all PGLs.^1^^,^^2^ In contrast, PGLs arising below the head and neck can arise anywhere along the sympathetic chain from the base of the skull to the bladder and prostate gland; most occur at the Organ of Zuckerkandl, a small collection of chromaffin cells on the abdominal aorta. Genitourinary PGLs represent approximately 6.7% of all PGLs, with the majority occurring in the bladder.^3^ Recent systematic reviews and larger case series have focused on larger, symptomatic primary prostatic paraganglioma, where our case focuses on a microscopic, incidentally discovered, intraprostatic inclusion with surrounding prostate adenocarcinoma. Our goal is to expand the documented phenotypes and highlight implications for diagnosis and management of suspected prostatic PGLs.

## 2 Case presentation

### 2.1 Patient course

A 63-year-old African-American man with a history of asthma, hypertension, morbid obesity, and glaucoma initially presented with a diagnosis of Grade Group 3 (GG3) prostate adenocarcinoma after transperineal (TP) fusion biopsy on 3/19/24. In October of 2024, his PSA was measured at 4.1 ng/mL. The patient denied episodes of headaches, palpitations, diaphoresis, and hematuria.

After thorough counseling about treatment options, the patient elected to undergo robotic-assisted laparoscopic radical prostatectomy with bilateral pelvic lymphadenectomy. Pathologic evaluation reported stage pT2N0MxR0 adenocarcinoma. Postoperatively, he reported erectile dysfunction and stress incontinence. In January of 2025, the patient's PSA was <0.1 ng/mL, confirming no evidence of biochemical recurrence.

After additional stains returned an addendum to the prostatectomy pathology report confirmed the finding of an incidental intraprostatic PGL measuring 1.5mm in the greatest dimension in the R base peripheral zone. Subsequent blood tests for plasma free metanephrines revealed normal levels (normetanephrine 46.1pg/mL and metanephrine 31.5pg/mL). The patient subsequently elected for surveillance of the PGL. He declined further imaging with a Ga-68 Dotatate Positron Emission Tomography (PET) scan. In his most recent visit, the patient has remained largely asymptomatic with reports of only mild urinary leakage requiring a thin pad. Of note, no preoperative plasma free metanephrines were obtained due to the lack of presenting patient symptoms that would be suggestive of catecholamine excess. This with the lack of functional imaging and germline genetic testing limits our definitive classification of the tumor function as well as assessment for hereditary PGL syndromes.

## 3 Discussion

The combined annual incidence of pheochromocytoma and PGL in the US is between 500 and 1600 cases, with the vast majority that occur outside of the adrenal medulla arising in the para-aortic region, followed by the bladder, mediastinum, head, and neck.^4^ Intraprostatic PGL is an exceptionally rare neoplasm, with fewer than fifteen cases reported in the literature to date. Often prostatic PGLs are localized, with some presenting with functional characteristics, such as producing catecholamines and causing hypertension or paroxysmal symptoms. The remainder present with lower urinary tract symptoms or are incidental findings. Prognosis is generally great when tumors are small, organ-confined, low-grade, and completely resected, but larger size, nodal involvement, highly proliferative, or adverse histology have higher risk.^1^^,^^3^, ^4^, ^5^ These tumors, which arise from the neural crest cells within the prostate, present unique diagnostic challenges due to their subtle presentation and often incidental discovery. In a study conducted by the Mayo Clinic of 290 PGLs analyzed, roughly one-third of those located below the neck were unexpected findings on pathology following surgery for an indeterminate mass, similar to our patient.^2^

The clinical presentation for affected patients depends on the functional status of the tumor as catecholamine or non-catecholamine secreting. Therefore, 24-h urinary total catecholamine and vanillylmandelic acid levels are a potential diagnostic tool, though this is dependent on an active functional status of the tumor. The 2025 NCCN clinical practice guidelines in oncology continue to recommend biochemical testing for PCC/PGL with plasma free metanephrines along with 24-h urinary fractionated catecholamines and metanephrines to confirm the diagnosis, acknowledging both potential false-positives and intermittent secretion, and to guide preoperative α-adrenergic blockade, cardiac evaluation, and intraoperative hemodynamic management.^4^ The most common presenting symptoms for nonfunctional prostate PGLs include hematuria, hemospermia, and difficult urination and defecation. Meanwhile, functional tumors can present with symptoms of hypertension, palpitation, headaches, dizziness, syncope, anxiety, and sweating, with symptom onset precipitated by micturition, defecation, or digital rectal examination.^5^ A major clinical consideration in diagnosing PGLs is the potential for catecholamine hypersecretion, resulting in resistant hypertension. Previous case reports have identified a link between paroxysmal hypertension crises and these tumors.^6^

Throughout the clinical course of our patient, none of these classical symptoms were present. The patient has had hypertension since 2013, prior to the diagnosis of his prostatic PGL. Throughout his clinical course for his PGL, his blood pressure has not altered post-surgery, with consistent readings of systolic pressure >150. The presentation of prostatic PGL on CT or MRI is similar to pheochromocytomas of other sites. In cases where the biochemical evidence is inconclusive, imaging studies such as I^123^-MIBG Scintigraphy or Ga-68 DOTATATE PET/CT scans can be helpful in characterizing these tumors.^7^ Of note, previous studies have indicated a lower sensitivity of I^123^-MIBG scintigraphy for PGLs as compared to pheochromocytomas.^7^ Other forms of imaging, such as contrast-enhanced CT and pelvic ultrasound, have also been described as a tool in diagnosis.^6^

Giannakodimos et al. recently conducted the largest systematic review to date of 25 patients with primary prostatic paragangliomas Their analysis revealed a heterogeneous spectrum of clinical presentations, with urinary symptoms (52%), blood loss (44%), and catecholamine-related symptoms (36%) among the most commonly reported manifestations. Biochemical testing was frequently abnormal when performed, and management strategies ranged from local excision to radical prostatectomy. This review underscores the variability in presentation and diagnostic pathways for prostatic paraganglioma.^8^

Compared with many cases summarized by Giannakodimos et al., our report describes a distinctly incidental, microscopic, nonfunctional lesion identified exclusively on final pathology in a patient undergoing prostatectomy for adenocarcinoma, further broadening the documented clinical and pathological spectrum of this rare entity.

**Fig. 1:**
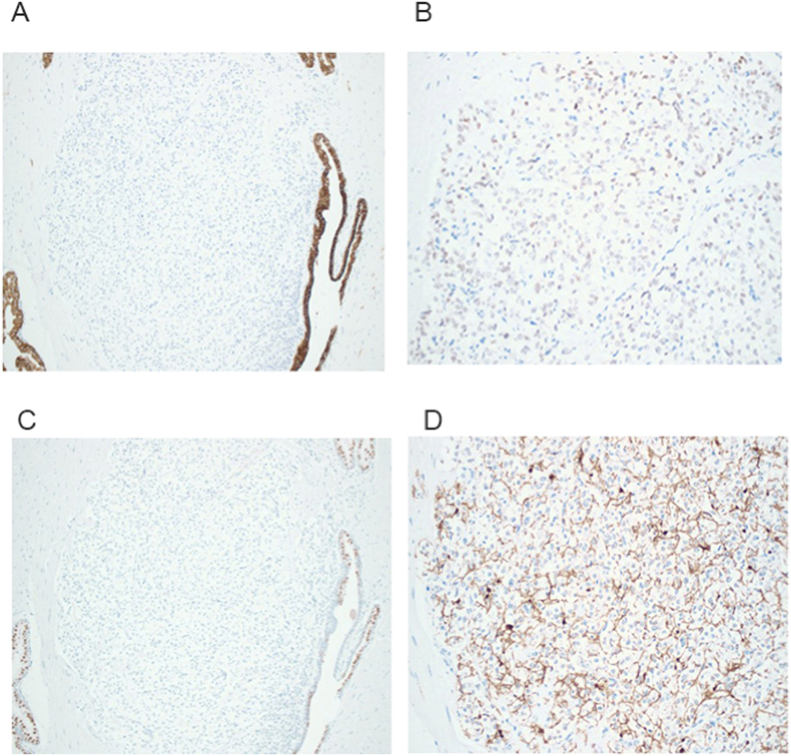
*Pathology stains.***A**) Cytokeratin AE1/AE3 (10 × ): Immunohistochemistry highlighted strong cytoplasmic staining in benign prostate glands, while paraganglioma cells demonstrated no expression; **B**) GATA-3 (20x) demonstrated weak nuclear expression within the tumor cells; **C**) NKX3.1 (10 × ): Paraganglioma cells were negative, whereas the adjacent benign prostate glands exhibited nuclear positivity; **D**) S100 protein (20 × ): Intense nuclear and cytoplasmic reactivity was highlighted in the sustentacular cells at the periphery of the tumor nests, supporting the characteristic staining pattern in paraganglioma.

**Fig. 2:**
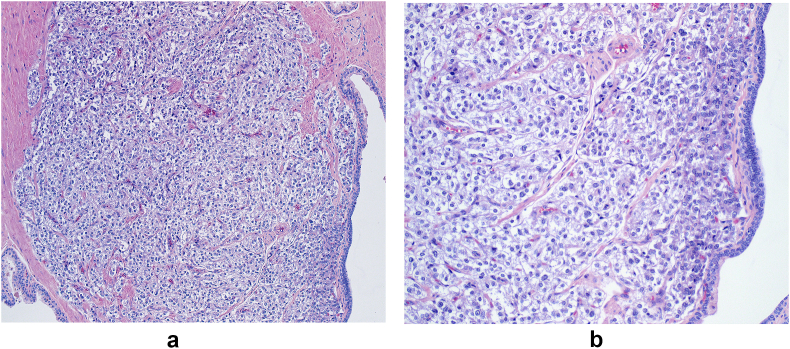
H&E stains show the characteristic nested (zellballen) growth pattern of tumor cells in a fibrovascular stroma.

In terms of diagnostic imaging, Wang et al. describe their case along with six others in the literature of prostatic PGL diagnosed on biopsy, including one via CT-guided biopsy, four through ultrasound guidance, and one by transurethral biopsy.^9^ However, as seen in our patient, the diagnosis can also be made as an incidental finding post-prostatectomy. This poses a diagnostic challenge, particularly for nonfunctional tumors in patients without other prostate pathologies, such as adenocarcinoma. In cases like ours, where there are no overt symptoms prompting evaluation, the tumor may remain undetected until after surgery. IHC analysis is essential to confirm the diagnosis and differentiate it from adenocarcinoma. As with other neuroendocrine tumors, prostatic PGLs are often immunoreactive for chromogranin, synaptophysin, neuron specific enolase, CD56, and S-100.^10^ Furthermore, the absence of cytokeratin staining, as in our patient, is used to differentiate PGLs from other neuroendocrine tumors such as carcinoid tumors. GATA-3 and tyrosine hydroxylase are additional markers that can aid in diagnosis. However, a negative GATA-3 result does not rule out the diagnosis, as sensitivity ranges from 70% to 90%^11^ (Fig. 1A–D). Representative H&E sections are shown in Fig. 2A–B.

Curative treatment for prostate PGL remains surgical resection. This is usually in the form of radical prostatectomy; however, successful cases have been reported of transurethral resection and prostate-sparing resection.^9^ Management of metastatic disease may involve chemotherapy and palliative radiotherapy. Yet, only four cases of metastatic prostate PGL have been reported in the English literature, reflecting its rarity and leaving the prognosis poorly characterized.^6^^,^^12^

As in our patient, the majority of prostate PGLs are benign, with no histologic criteria currently defined to differentiate between the two. Tumor metastasis and vascular invasion are the modalities used to define a metastatic lesion over a benign one.^12^ Therefore, our patient was diagnosed with a benign PGL based on the absence of metastatic lesions, although he declined full-body cross sectional imaging. To date, only five cases of malignant prostatic PGL have been reported, with metastatic spread observed in various sites. These include the bladder and seminal vesicles in one case, three left iliac nodes in another, the retroperitoneum in a third, the bone in a fourth, and in the fifth case, extensive metastasis involving the bladder, rectum, seminal vesicles, and multiple pelvic lymph nodes.^10^^,^^12^, ^13^, ^14^, ^15^ In three of five cases, no recurrence was observed at the most recent follow-ups, which occurred at two months, six months, and five years, respectively. However, the fifth case involving a 17-year-old patient with extensive metastasis proved unresponsive to chemotherapy and was deemed unresectable during surgical exploration.

PGLs are frequently associated with inherited disorders, particularly the PGL syndromes (types 1-5), which result from autosomal dominant mutations in genes encoding different subunits of the mitochondrial enzyme succinate dehydrogenase (SDH). SDH plays a crucial role in both the mitochondrial electron transport chain and the Krebs cycle. In the former, it functions as an electron transporter to cytochrome *c* of complex III, while in the latter, it catalyzes the conversion of succinate to fumarate. Loss of SDH activity leads to succinate accumulation and increased reactive oxygen species production, which in turn drives the stabilization of hypoxia-inducible factors. As a result, tumors with SDH deficiency exhibit upregulation of hypoxia-responsive genes, contributing to their tumorigenic potential.^16^ These syndromes are characterized by an increased risk of developing PGLs, pheochromocytomas, renal cell carcinoma, gastrointestinal stromal tumors, and pituitary adenomas.^17^ Each subtype of PGL syndrome presents with varying cancer risks and distinct tumor locations based on mutation. Notably, approximately 40% of all PGLs have been linked to SDH deficiency.^18^ To date, only one reported case of PGL involving the prostate has been linked to an inherited genetic mutation. This case occurred in a 19-year-old male with a known heterozygous germline SDHB mutation, part of a familial PGL syndrome.^19^ However, the tumor originated in the bladder and subsequently invaded the prostate, rather than arising primarily in the prostate itself. This distinction suggests that, unlike PGLs in other locations, primary prostatic PGLs may not have a strong genetic component or may arise through different molecular mechanisms. Further research and inquiry are needed to establish if a link exists between prostatic PGL and the PGL syndromes, as patients with a PGL are recommended to receive genetic counseling upon diagnosis.^4^ Our patient underwent TEMPUS xT molecular profiling but no actionable somatic mutations were found, combined positive score (CPS) < 1, tumor mutational burden 2.1m/MB, and microsatellite stable. There were variants of unknown significance in PAX3, NOTCH1, and PBRM1, but none of these genes have been implicated in PGL or pheochromocytoma. NOTCH1 pathway dysregulation has been described in some neuroendocrine tumors but not specifically with PCC or PGL.^4^^,^^20^

## 4 Conclusion

We present a rare case of incidental intraprostatic PGL in a 63-year-old African American man who underwent robotic prostatectomy for adenocarcinoma. The gaps in the literature highlight the need for a more standardized approach to diagnosing and managing these tumors, as well as a more comprehensive understanding of their clinical implications, especially given the risks of hypertensive complications. The risk of hypertensive crises reported by previous case reports highlights the beneficial role of perioperative alpha blockade to mitigate this risk. In addition, post-operative monitoring of hypertension is advised. Intraprostatic PGL is a rare tumor that should be included in the differential diagnosis of prostate tumors despite their rarity as they present incidentally or asymptomatically in most cases. Their early diagnosis, monitoring, and intervention remain critical for favorable patient outcomes.

## CRediT authorship contribution statement

**Diego Gonzalez:** Writing – original draft, Writing – review & editing. **Francis Ryan:** Writing – original draft, Writing – review & editing. **Kris Kokoneshi:** Writing – original draft, Writing – review & editing. **Sam Kwon:** Writing – original draft, Writing – review & editing. **Liza Khutsishvili:** Writing – original draft, Writing – review & editing. **Fiona Wardrop:** Writing – original draft, Writing – review & editing. **Jacob Weiss:** Writing – original draft, Writing – review & editing. **Shawn Reginauld:** Writing – original draft. **Maher Ali:** Data curation, Formal analysis, Visualization. **Abiye Kassa:** Data curation, Formal analysis, Visualization. **Michael Whalen:** Conceptualization, Supervision, Writing – review & editing.
